# Evaluation of Plasma Concentrations of Galectins-1, 2 and 12 in Psoriasis and Their Clinical Implications

**DOI:** 10.3390/biom13101472

**Published:** 2023-09-30

**Authors:** Julia Nowowiejska, Anna Baran, Justyna Magdalena Hermanowicz, Beata Sieklucka, Dariusz Pawlak, Iwona Flisiak

**Affiliations:** 1 Department of Dermatology and Venereology, Medical University of Bialystok, Zurawia 14 St., 15-540 Bialystok, Poland; anna.baran@umb.edu.pl (A.B.); iwona.flisiak@umb.edu.pl (I.F.); 2 Department of Pharmacodynamics, Medical University of Bialystok, Mickiewicza 2C St., 15-089 Bialystok, Poland; justyna.hermanowicz@umb.edu.pl (J.M.H.); beata.sieklucka@umb.edu.pl (B.S.); dariusz.pawlak@umb.edu.pl (D.P.)

**Keywords:** galectin 1, galectin 2, galectin 12, gal-1, gal-2, gal-12, galectin, atherosclerosis, psoriasis, metabolic syndrome

## Abstract

Psoriasis is a complex disease that nowadays is considered not only a dermatosis but a kind of systemic disorder associated with many accompanying diseases. Metabolic complications leading to cardiovascular incidences are the cause of increased mortality in psoriatic patients. Galectins (gal) are beta-galactoside-binding lectins that exert different functions, including engagement in metabolic processes. Our aim was to assess the concentrations of gal-1, 2 and 12 in psoriatics, to establish their potential clinical implications, including in metabolic complications. Plasma galectins were assessed by ELISA in 60 psoriatic patients and 30 controls without dermatoses and a negative family history of psoriasis. Plasma concentrations of all galectins were significantly higher in patients than controls (gal-1 with *p* < 0.001, gal-2 and 12 with *p* < 0.05). There were no correlations between galectins concentrations and psoriasis severity in PASI or disease duration (*p* > 0.05). Gal-1 and 12 were significantly negatively correlated with GFR (*p* < 0.05, *p* < 0.01, respectively) and gal-2 with HDL (*p* < 0.05). Gal-2 was significantly positively correlated with CRP (*p* < 0.05) and gal-12 with fasting glucose (*p* < 0.01). Based on the results and given the reported role of galectins in metabolic disorders we may conclude that gal-1, 2 and 12 could be potentially engaged in metabolic complications in psoriatics, most probably in atherosclerosis. Gal-2 could be perhaps further investigated as a marker of metabolically induced inflammation in psoriasis, gal-1 and gal-12 as predictors of renal impairment in psoriatics due to metabolic disorders. Potentially, gal-12 could be considered in the future as a marker of carbohydrate metabolism disorders in psoriatics.

## 1. Introduction

Psoriasis is a complex disease that nowadays is considered not only a dermatosis but a kind of systemic disorder associated with many accompanying diseases [1]. To date, psoriasis has been linked to a number of comorbidities but certainly the most well-documented and the key one at the same time is the relationship between psoriasis and metabolic disorders [1]. Metabolic complications leading to cardiovascular incidences are the cause of increased mortality in psoriatic patients [2]. Psoriasis has been well linked to obesity, arterial hypertension, dyslipidemia, type 2 diabetes mellitus and non-alcoholic fatty liver disease [3]. The source of such wide comorbidity is the so-called ‘psoriatic march’. It has been suggested that systemic inflammation which occurs in psoriasis leads to insulin resistance and endothelial dysfunction, further resulting in atherosclerosis [3,4]. For this reason, research in this area has exploded and our team has also explored this topic.

Galectins are beta-galactoside-binding lectins and all of them possess at least one carbohydrate recognition domain [5,6]. Galectins are subdivided into three families: prototype, chimera type and tandem repeat type [7]. Depending on their tissue specificity and their localization, they exhibit different functions both intra- and extracellularly [6]. They are produced in the cytosol and then can be translocated to the nucleus, cell membrane or outside the cells [8]. Galectins are able to gather and form oligomers, which can further be reorganized allowing for alterations of their activity [6]. At least 12 types of galectins have been identified in the human body [6]. Based on the current knowledge, galectin-1, 2, 3, 9 and 12 are involved in metabolic disorders [9,10,11,12], and gal-3 and 9 have been fairly exhaustively studied.

Gal-1 is a prototype galectin [7], encoded by the *LGALS1* gene on chromosome 22q12 [13]. Gal-1 is secreted by keratinocytes, adipocytes, fibroblasts, lymphocytes and macrophages [13]. It is prominently expressed in endothelial cells and smooth muscles in arteries [9]. Gal-1 has been assigned various biological functions, such as involvement in immune response, inflammation, cell proliferation and angiogenesis [13]. Of note, from the dermatological point of view, gal-1 has been suggested to participate in the regulation of keratinocyte proliferation [13] and influence the immune response in several autoimmune diseases [6].

Gal-2, similar to gal-1, is a prototype galectin encoded by the *LGALS2* gene on chromosome 22 and appears in the form of a dimer [7,8,14]. It is expressed in the gastrointestinal tract, cardiovascular system and placenta [8]. It is engaged in immune response, angiogenesis, inflammatory processes, cancerogenesis and has pro-apoptotic properties [8,14].

Gal-12 belongs to the tandem repeat type family of galectins [7] and is encoded by the *LGALS12* gene on chromosome 11 [15]. It is expressed mostly in the adipose tissue and peripheral leukocytes, especially macrophages, but also in the heart, spleen or pancreas [16]. It is found in lipid droplets and exhibits great specificity for adipocytes [15]. The biological role of gal-12 is the regulation of lipolysis via the influence on lipolytic protein kinase A (PKA) [15]. Gal-12 is also involved in cell death, proliferation and differentiation [14,16]. As for the skin, gal-12 affects the proliferation and differentiation of sebocytes and regulates skin immune response [11].

The three galectins we selected are also involved in different metabolic disorders, namely atherosclerosis, carbohydrate metabolism disorders or dyslipidemia [9,10,15]. Their engagement in such disorders makes them interesting objects for research as potential biomarkers or therapeutic targets. Hence, we decided to investigate them in psoriasis, especially since gal-2 and 12 have never been studied in this disease. So far, only gal-1, 3, 7, 8 and 9 have been studied in psoriasis, either in blood or tissue or both [17,18,19,20,21].

## 2. Materials and Methods

The study enrolled 60 patients (21 women and 39 men) with a flare of plaque psoriasis, with a mean age of 49 ± 2.3 years old. Patients were compared to 30 sex- and age-matched volunteers without dermatoses and a negative family history of psoriasis. All participants signed an informed consent before initiation. There were the following exclusion criteria from the study applied: age under 18 years old, pregnancy, types of psoriasis other than plaque, dietary restrictions, intake of oral medications at least 3 months prior to the study, infectious diseases, autoimmune diseases (other than psoriasis) and malignant neoplasms. The severity of psoriatic skin lesions was assessed by the Psoriasis Area and Severity Index (PASI) and was performed by the same dermatologist in every patient. The group of patients was divided into three subgroups depending on the severity of the disease: PASI I (PASI < 10)—mild psoriasis, PASI II (PASI 10–20)—moderate psoriasis and PASI III (PASI > 20)—severe psoriasis. Patients were also divided into two subgroups according to psoriasis duration: more or less than 15 years. Body mass index (BMI) was calculated as weight/height^2^. Laboratory tests, including C-reactive protein (CRP), complete blood count, plasma fasting glucose, lipid parameters, aminotransferases, creatinine and urea were performed before the study. The study was approved by the Bioethics Committee of the Medical University of Bialystok (number: APK.002.19.2020) and was conducted in accordance with the principles of the Helsinki Declaration [22].

### 2.1. Plasma Collection

Fasting blood samples were taken using vacuum tubes. They were centrifugated for 10 min at 2000× g. The obtained plasma was stored at −80 °C until further analysis. Laboratory parameters were measured using routine techniques. Gal-1,2 and 12 plasma concentrations were measured with an enzyme-linked immunosorbent assay (ELISA) provided by Cloud Clone^®^ (Houston, TX, USA; SEA321Hu, SEA302Hu, SEA312Hu). Optical density was read at a wavelength of 450 nm. The concentrations were assessed by interpolation from calibration curves prepared with standard samples provided by the manufacturer. All laboratory tests were performed by the same person in standardized laboratory settings.

### 2.2. Statistical Analysis

Shapiro–Wilk’s W test of normality was used for data distribution analysis. The normally distributed data were analyzed using Student’s t-test or one-way analysis of variance (ANOVA) and shown as mean ± SD. The non-Gaussian data were presented as median (full range) and analyzed using the non-parametric Mann–Whitney test or Kruskal–Wallis test. The relationships between the examined parameters were assessed with Spearman’s rank test. Statistical analysis was conducted using GraphPad Prism 9.4 software. The differences were deemed statistically significant when *p* < 0.05.

## 3. Results

Basic characteristics of the patients and the control group are presented in Table 1.

There were no significant differences between patients and controls in terms of gender, age or BMI (NS).

### 3.1. Galectin 1

**Figure 1:**
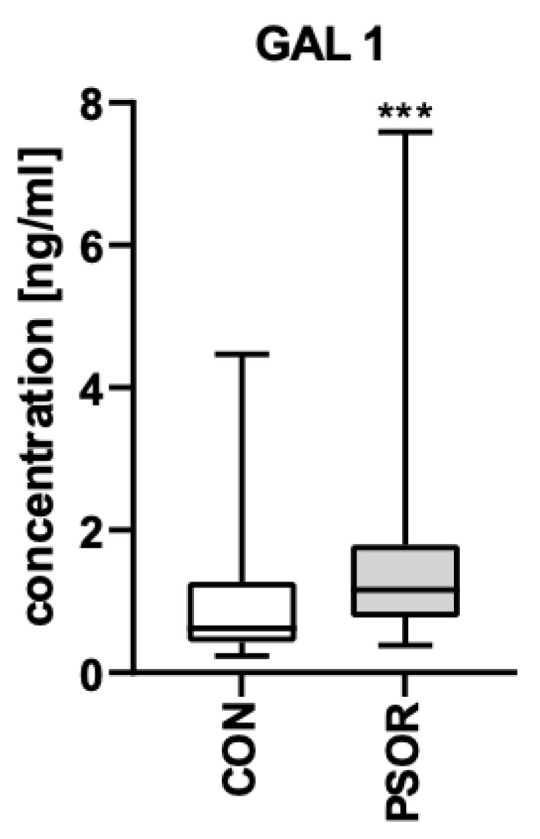
Plasma concentration of gal-1 in patients vs. controls. *** means a statistically significant difference compared to the control group with *p* < 0.001.

Plasma concentration of gal-1 was significantly higher in patients than controls (*p* < 0.001) (Figure 1).

**Figure 2:**
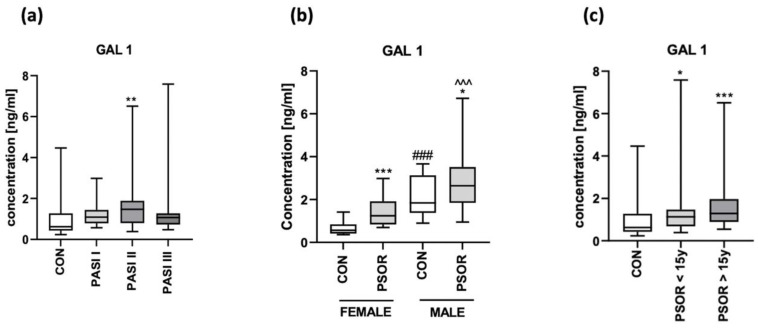
Plasma gal-1 concentration in patients after division according to PASI (**a**), sex (**b**), and psoriasis duration (**c**), compared to controls. */**/*** means a statistically significant difference compared to the control group with *p* < 0.05/0.01/0.001, respectively; ### means a statistically significant difference compared to the female controls with *p* < 0.001; ^^^ means a statistically significant difference compared to the psoriatic females with *p* < 0.001.

After the division of patients according to psoriasis severity expressed by PASI, there were no differences in gal-1 concentration between the subgroups (NS) (Figure 2a).

After the division of patients according to their gender, we observed significantly higher gal-1 concentrations in psoriatic men compared to women (*p* < 0.001) (Figure 2b). After the division of patients according to the duration of psoriasis, we did not find any significant difference in gal-1 concentrations between the subgroups (Figure 2c).

**Figure 3:**
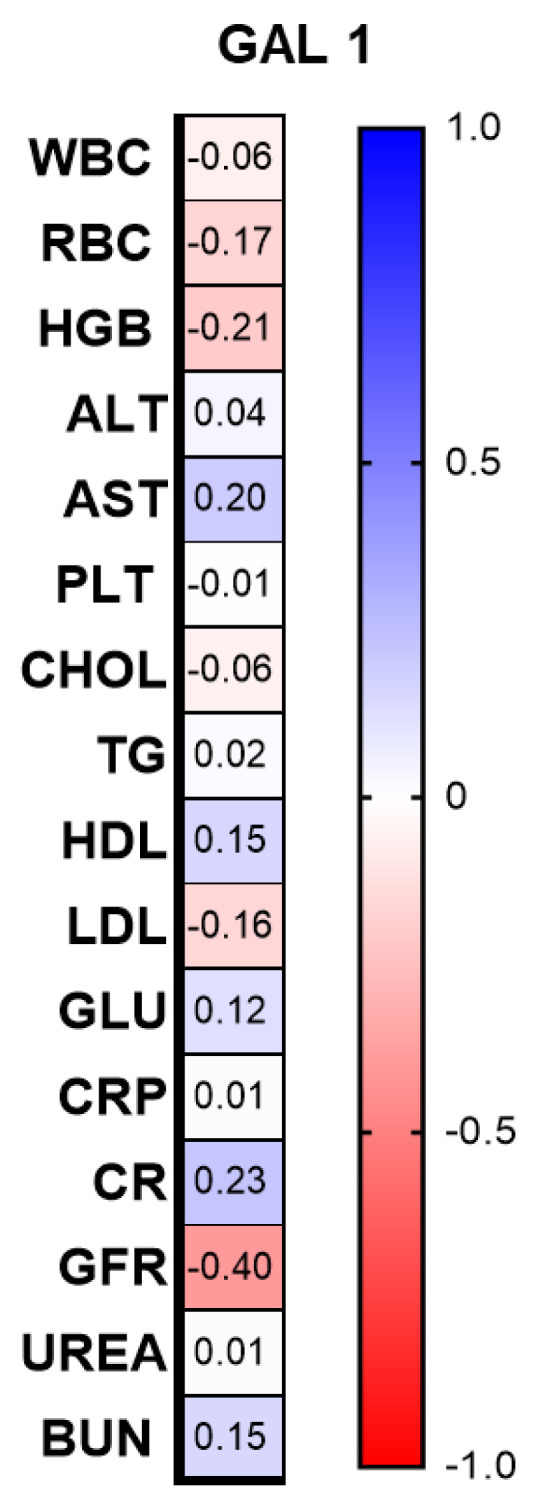
Correlations between the laboratory parameters and gal-1 concentrations. TGs, triglycerides; ALT, alanine aminotransferase; AST, asparagine aminotransferase; GLU, fasting glucose; Chol, total cholesterol; HDL, high-density lipoprotein; LDL, low-density lipoprotein; RBC, red blood cells; WBC, white blood cells; PLT, platelets; HGB, hemoglobin; CR, creatinine.

Among the correlations with laboratory parameters, we found a significant negative correlation between gal-1 and GFR (R = −0.4; *p* = 0.018) and an upward trend towards creatinine concentration (*p* = 0.08) (Figure 3).

Gal-1 was significantly positively correlated with the age of patients (R = 0.28; *p* = 0.028). There were no direct correlations between gal-1 concentrations and PASI, BMI and psoriasis duration (NS).

### 3.2. Galectin 2

**Figure 4:**
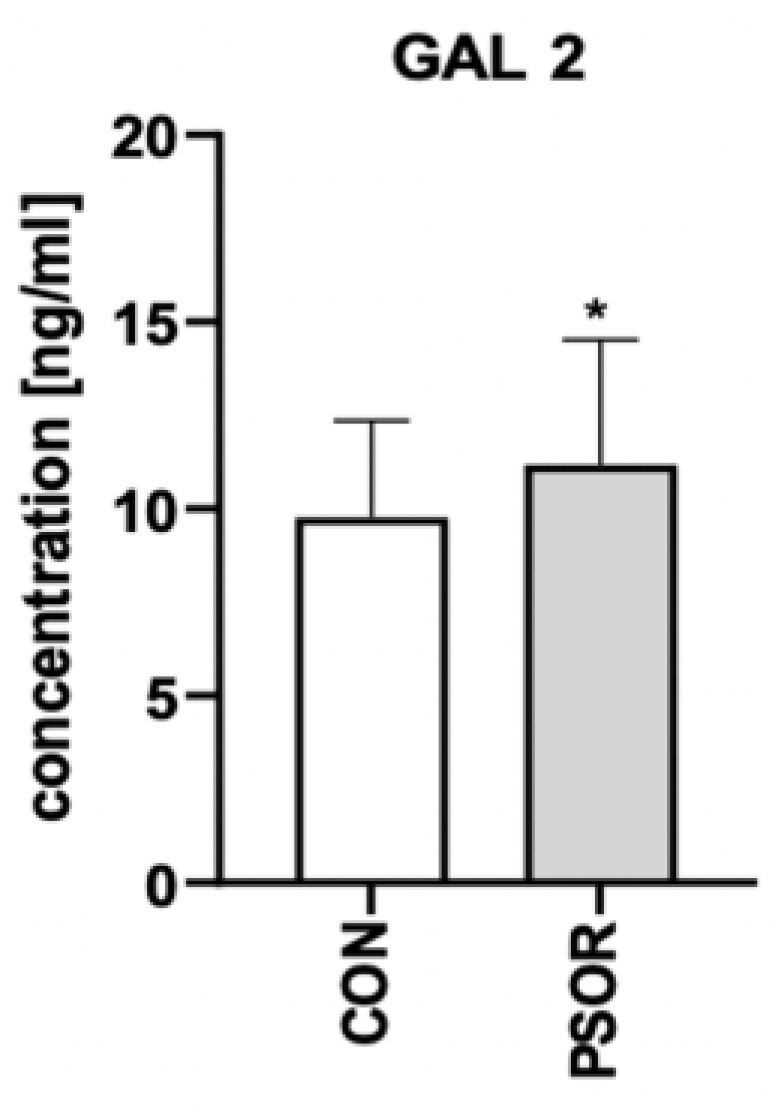
Plasma concentration of gal-2 in patients vs. controls. * means a statistically significant difference compared to the control group with *p* < 0.05.

Plasma concentration of gal-2 was significantly higher in patients than controls (*p* < 0.05) (Figure 4).

**Figure 5:**
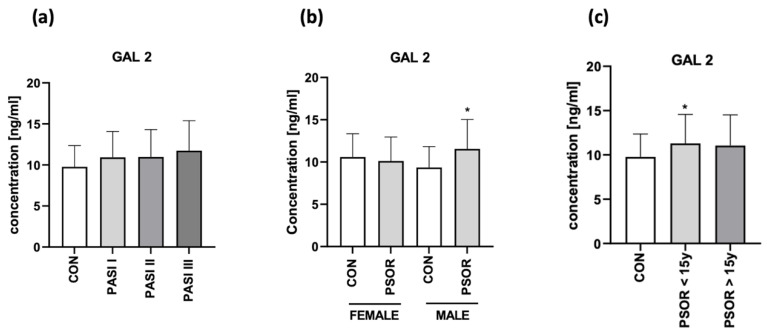
Plasma gal-2 concentration in patients after division according to PASI (**a**), sex (**b**), and psoriasis duration (**c**), compared to controls. * means a statistically significant difference compared to the control group with *p* < 0.05.

After the division of patients according to psoriasis severity expressed by PASI, sex or psoriasis duration, there were no differences in gal-2 concentration between the subgroups (NS) (Figure 5a–c).

**Figure 6:**
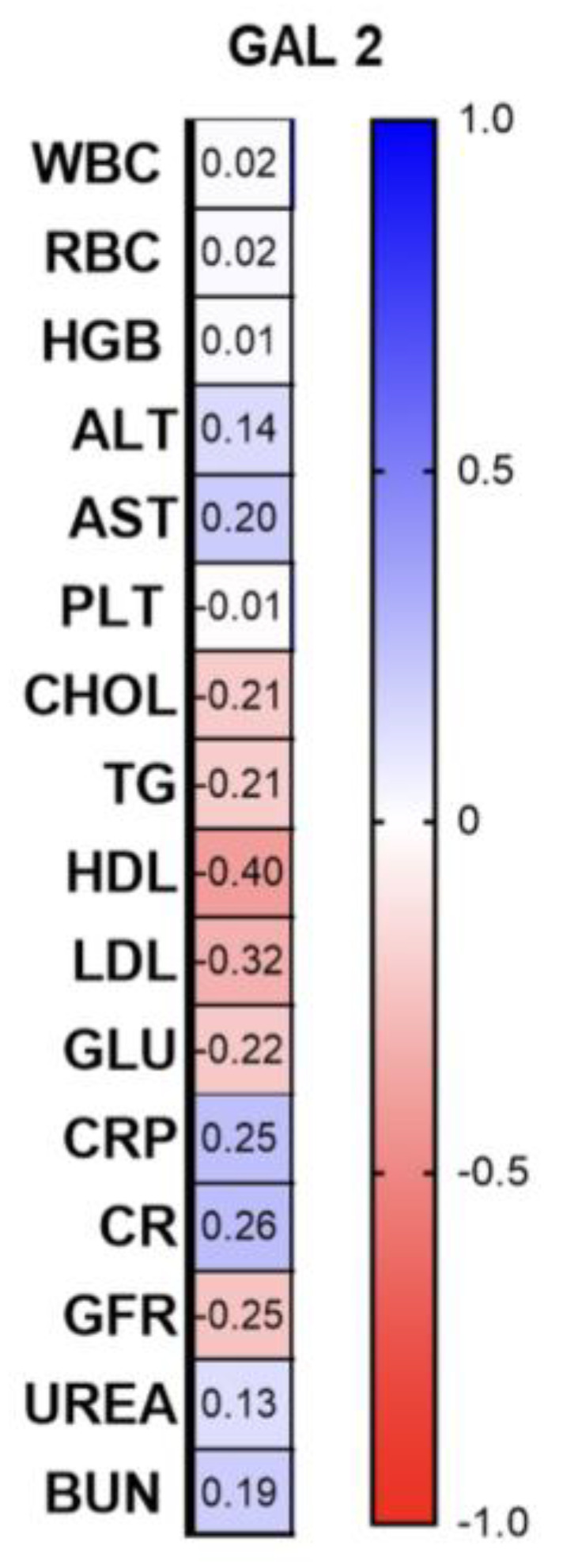
Correlations between the laboratory parameters and gal-2 concentrations. TGs, triglycerides; ALT, alanine aminotransferase; AST, asparagine aminotransferase; GLU, fasting glucose; Chol, total cholesterol; HDL, high-density lipoprotein; LDL, low-density lipoprotein; RBC, red blood cells; WBC, white blood cells; PLT, platelets; HGB, hemoglobin; CR, creatinine.

Among the correlations with laboratory parameters, we found a significant negative correlation between gal-2 and HDL (R = −0.4; *p* = 0.02) and a positive with CRP (R = 0.25; *p* = 0.046) (Figure 6).

There were no direct correlations between gal-2 concentrations and PASI, BMI, age, gender of patients and psoriasis duration (NS).

### 3.3. Galectin 12

**Figure 7:**
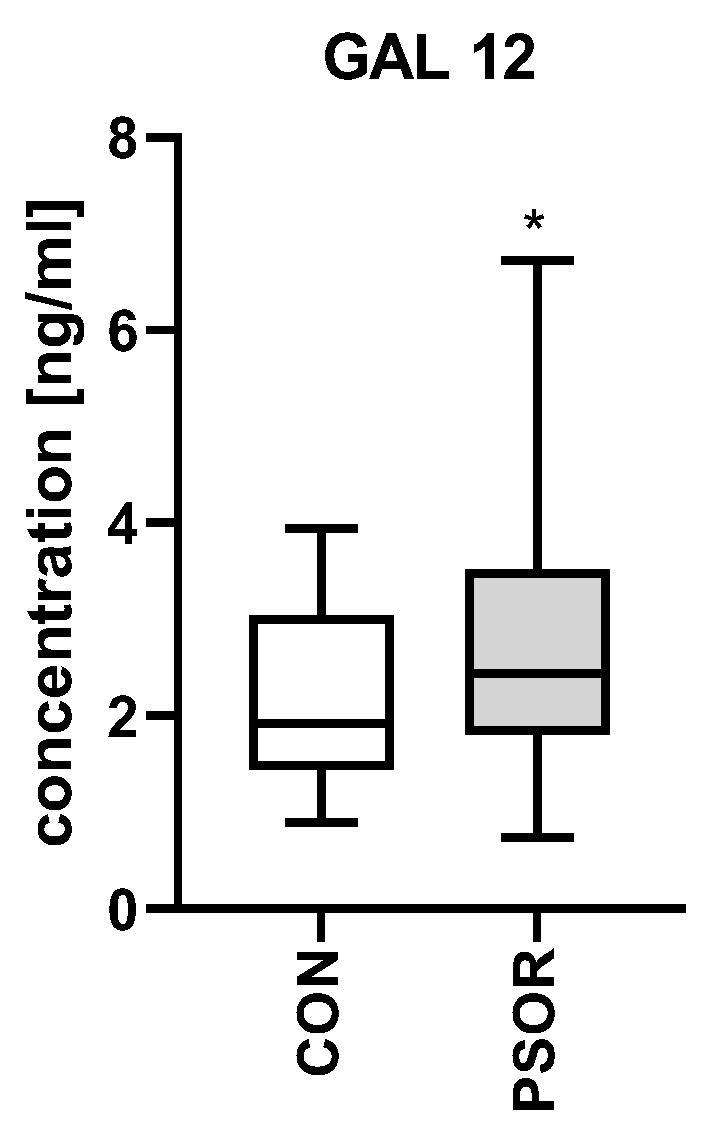
Plasma concentration of gal-12 in patients vs. controls. * means a statistically significant difference compared to the control group with *p* < 0.05.

Plasma concentration of gal-12 was significantly higher in patients than controls (*p* < 0.05) (Figure 7).

**Figure 8:**
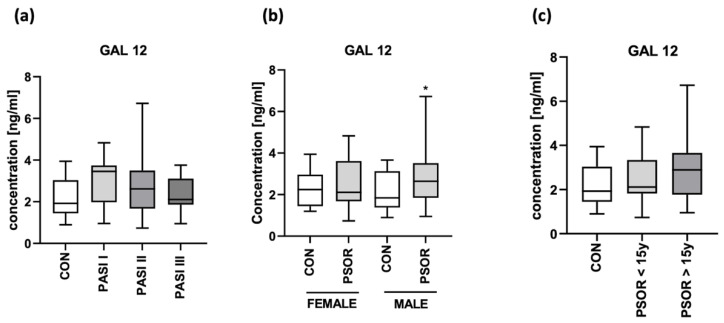
Plasma gal-12 concentration in patients after division according to PASI (**a**), sex (**b**), and psoriasis duration (**c**), compared to controls. * means a statistically significant difference compared to the control group with *p* < 0.05.

After the division of patients according to psoriasis severity expressed by PASI, sex or psoriasis duration, there were no differences in gal-12 concentration between the subgroups (NS) (Figure 8a–c).

**Figure 9:**
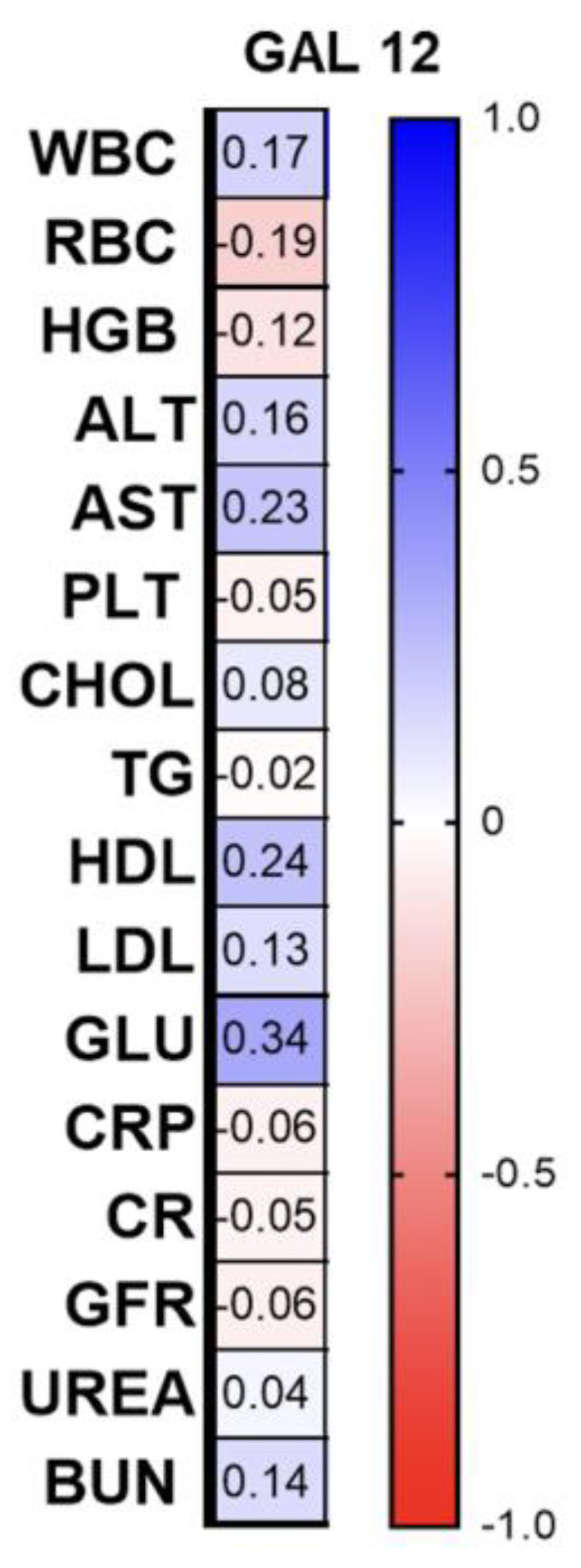
Correlations between the laboratory parameters and gal-12 concentrations. TGs, triglycerides; ALT, alanine aminotransferase; AST, asparagine aminotransferase; GLU, fasting glucose; Chol, total cholesterol; HDL, high-density lipoprotein; LDL, low-density lipoprotein; RBC, red blood cells; WBC, white blood cells; PLT, platelets; HGB, hemoglobin; CR, creatinine.

Among the correlations with laboratory parameters, we found a significant positive correlation between gal-12 and fasting glucose (R = 0.34; *p* = 0.009) and a negative with GFR (R = −0.06; *p* = 0.0018) (Figure 9).

Gal-12 was significantly positively correlated with the age of patients (R = 0.32; *p* = 0.014). There were no direct correlations between gal-12 concentrations and PASI, BMI and psoriasis duration (NS).

## 4. Discussion

Galectins have already gained the attention of scientists, including with regard to psoriasis; however, considering the variety of this group, there is still much to discover. So far, the best-studied galectin in psoriasis remains gal-3. It has been proven to be elevated in sera of psoriatics in most studies, including ours [17,23,24].

Galectins have been also evaluated with regard to other skin diseases, especially atopic dermatitis (AD). Regarding these galectins that we chose in this experiment, the protein and mRNA of gal-1 were reported by Correa et al. to be increased in patients with AD [25] and in another study by the same group, recombinant gal-1 was successfully used as a therapeutic agent in the AD induced in mice [26]. Gal-1 has been also assessed in systemic sclerosis and suggested to act as a protective factor against digital vasculopathy [27]. Gal-2 has been studied in contact allergic dermatitis [28] and wound healing [29]. Gal-12, on the other hand, has been proven to simulate IL-4 signaling in sebaceous glands and the development of AD phenotype in mice [11].

Atherosclerosis and psoriasis have much in common. Both depend on chronic inflammation and there are similarities between psoriatic and atherosclerotic plaque with regard to cells engaged in these diseases and the cytokine profile [30]. This is why we became interested in the role of selected galectins, with proven engagement in atherogenesis, in psoriasis. Nevertheless, we also looked into other metabolic disorders, as they mutually influence each other with psoriasis, and psoriatic patients have a documented increased risk of developing such complications.

The first of the galectins that we studied, gal-1, is the best-characterized galectin and has already been studied in psoriasis; however, the aims of the authors were different from ours and analyzed from an immunological point of view [20]. In the very recent paper by El Bably et al. from 2023, the goal was to distinguish between rheumatoid arthritis (RA), psoriatic arthritis (PsA) and solely psoriasis based on gal-1 assessment [20]. They found that gal-1 was significantly higher in patients with RA compared to PsA or psoriasis [20]. Gal-1 has been also assessed in psoriatic tissue and found to be increased in the dermis and epidermis compared to controls in one study [31], but decreased, along with lower mRNA expression in two others [20,31,32].

In our study, we noticed a significantly higher plasma concentration of gal-1 in psoriatic patients compared to controls without dermatoses indicating its role in this specific group of patients.

Gal-1 has been implicated in the pathogenesis of various metabolic disorders. First, it is involved in atherosclerosis. Gal-1 has been found to be upregulated in vascular smooth muscle cells (VSMCs) and monocytes. It has been suggested that gal-1 plays a role in the proliferation, adhesion and migration of these cells [33]. That, on the other hand, is an important step in the development of atherosclerotic plaque [9]. VSMC migration is essential for fibrous cap formation due to the release of their components such as elastin and collagen [6]. Moreover, gal-1 has been studied in atherosclerotic plaques of mice and found to be weakly expressed in intimal plaques and media of atherosclerotic aortas. At the same time, therapy with atorvastatin did not alter the expression of gal-1 in these vessels [33]. Further evidence for gal-1 participation in atherosclerosis is the relationship with lipoprotein A (LpA). LpA is able to bind in situ to tissue gal-1 [9]. Inflammation of the blood vessel walls may promote gal-1 overexpression, which may further result in lipoprotein retention, and LpA accumulation is engaged in atherogenesis [9]. As for the clinical implications of atherosclerosis, serum gal-1 has been found to be elevated after 4 weeks from the ischaemic stroke [34].

The second metabolic disorder that gal-1 is associated with, and is the basis of metabolic syndrome at the same time, is obesity. Blood concentrations of gal-1 have been associated with obesity and found to be even positively correlated with BMI and plasma triglyceride concentrations, but negatively with plasma HDL [35]. Gal-1 is also related to leptin, which is a hormone increasing hunger and lipolysis, at the same time decreasing blood insulin levels [36]. In studies on obese mice, gal-1 knockout resulted in lower serum levels of leptin, similar to the inhibition of gal-1 by oral administration of thiodigalactoside [35]. Unfortunately, in our research, we did not observe any correlation between plasma gal-1 and BMI or lipid parameters, so probably they are not directly related in this particular group of patients.

Another component of metabolic syndrome is type 2 DM. Elevated concentrations of gal-1 have been found in individuals suffering from DM and this galectin has even been proposed as a new marker of this disease and its risk factor [35]. Alas, in our study, we did not find a direct correlation between plasma gal-1 and glycemia.

In this experiment, we found a negative correlation between plasma gal-1 and GFR, as well as an upward trend toward creatinine concentration. Elevated plasma gal-1 levels have been already reported in a small sample of patients with diabetic nephropathy [37] and suggested as predictors of kidney impairment, independently of diabetes and other risk factors, e.g., in subjects after coronary angiography [38]. However, surprisingly, there are studies that show the protective influence of gal-1 on renal pathology [35]. Considering our results and data obtained by other scientists, gal-1 could probably be a predictor of renal impairment in psoriatics due to metabolic disorders, which obviously affect kidneys.

Analyzing different clinical aspects of our patients, we did not observe a direct correlation between gal-1 concentrations, suggesting increased metabolic burden, according to the skin lesions severity or psoriasis duration. In comparison, in the study by ElBably et al., skin gal-1 did not correlate with PASI or BSA either [20]. Importantly, gal-1 turned out to be significantly higher in male patients compared to female ones highlighting the higher possibility of metabolic complications in men, including psoriatics.

As far as we are concerned, there are no studies on the use of gal-1 inhibitors in psoriasis.

Gal-2 is much less studied in general and has never been assessed in psoriasis. As for metabolic disorders, gal-2 expression was found to be correlated with a low arteriogenic response in patients with coronary artery disease [10]. BRCA1-associated protein (BRAP) has the ability to bind gal-2 and it has been shown that BRAP is upregulated in VSMCs and macrophages of atherosclerotic plaques in some populations, whereas single-nucleotide polymorphism in the *BRAP* gene is associated with an increased risk of myocardial infarction (MI) [10]. Moreover, a polymorphism in the *LGALS2* gene has been related to the severity of coronary atherosclerosis, but contrary to *BRAP*, not with MI [10,39]. Gal-2 has also the ability to induce inflammation in VSMCs [40]. Another clue for gal-2 participation in atherosclerosis development is that drugs inhibiting this galectin have been suggested as a future potential treatment option [10]. In the experiment by Kane et al. after treating the mice with anti-gal-2 nanobodies, there was a reduction in atherosclerotic burden, namely slower progression and smaller area of the atherosclerotic plaques [41]. Another beneficial effect of this therapy was lowering the plasma cholesterol which is obviously a risk factor for atherosclerosis [41]. As for glucose metabolism impairment, in one study it has been observed that fasting plasma glucose and serum insulin were statistically significantly associated with *LGALS2* rs7291467, and it was independent of BMI and waist-hip ratio (WHR); this experiment, however, was conducted only in women [40].

In our study, we observed significantly higher gal-2 concentrations in psoriatic patients compared to controls, which could again point to a higher risk of metabolic complications in psoriasis. Gal-2 concentrations were not dependent on any clinical-demographic variables such as sex, age, skin lesions severity or duration and there is no other study in which gal-2 would be assessed with reference to such parameters. Apparently, they do not affect significantly gal-2 plasma concentration. However, we found a negative correlation between gal-2 and HDL concentration which is consistent with its role in the promotion of dyslipidemia, as well as a positive correlation with CRP. It has been documented that gal-2 induces the production of lymphotoxin-a, which has pro-inflammatory properties; therefore, gal-2 also has the ability to indirectly promote tissue inflammation [8]. Gal-2 could be perhaps further investigated as a marker of metabolically induced inflammation in psoriasis.

However, there have been attempts to use gal-2 inhibitors, and to the best of our knowledge, there are no studies on their application in psoriasis.

The last galectin assessed in this study was gal-12, which, similar to gal-2, has never been evaluated in psoriatic patients. In this experiment, we found a significantly increased concentration of plasma gal-12 compared to controls. Its main action is the negative regulation of lipolysis [42]. Gal-12 downregulates cAMP which usually activates PKA. Therefore, PKA cannot promote triglycerides hydrolysis [43]. The potential role of this galectin has been also suggested based on what happens under the condition of its deficiency. First, the knockdown of gal-12 prevents foam cell formation in human macrophages [42]. Reduction in gal-12 also leads to lower adipocytes differentiation [44] and mice lacking gal-12 have lower adipose tissue content [45]. In instances lacking gal-12, it improves sensitivity to insulin and glucose tolerance in overweight animals and down-regulates secretion of proinflammatory cytokines (TNFα, IL-6) [42]. In the case of insulin resistance associated with hyperglycemia, increased secretion of vasoconstrictive mediators is observed which leads to endothelial dysfunction. On the other hand results in impaired secretion of nitric oxide which causes vasodilation, which altogether are the components of atherosclerosis development. It has been suggested that gal-12—by affecting sensitivity to insulin—also indirectly influences the endothelium function in this way [42]. This data point to the prominent role of gal-12 in obesity, atherosclerosis and carbohydrate metabolism disorders pathogenesis.

In psoriatic patients, we observed a positive correlation between gal-12 and fasting glucose. Potentially, gal-12 could be considered in the future as a marker of carbohydrate metabolism disorders in psoriatics. Moreover, similar to gal-1, it was negatively correlated with GFR which may implicate its value as a renal function marker in psoriatics with metabolic complications.

Gal-12 has been suggested as a therapeutic target in insulin resistance, type 2 DM and other metabolic disorders [15]. Hsu et al. investigated the role of corn silk extract and β-sitosterol as gal-12 inhibitors in mice and observed weight loss and reduction in the number of adipocytes in liver and adipose tissue [46]. The administration of β-sitosterol has already been attempted in psoriasis years ago but with no evident beneficial effect [46]. The blood cholesterol levels fluctuated and there was no relevant clinical improvement of skin lesions [46]. However, it should be noted that the experiment was performed on a very small sample [46]. On the other hand, in a more recent study, after the application of methanolic extract from the plant *Andrographis nallamalayana* on the imiquimod-induced psoriatic skin in mice, there was an improvement in skin condition [47]. However, it must be highlighted that in this study a multi-component agent and not a pure β-sitosterol was applied. In the most recent study from 2023, again on mice, after application of β-sitosterol there was a marked reduction in epidermal hyperplasia and cell infiltrations [48]. Such observations could support the data on gal-12 involvement in psoriasis.

Gal-1 and gal-12 were positively correlated with the age of patients which emphasizes the greater risk of metabolic complications in older age, including in psoriasis.

## 5. Limitations and Future Directions

The main limitation of our study is not enough diverse group of participants with high male predominance, originating from one city and of one ethnicity. In the future, we plan to assess the tissue expression of gal-1, 2 and 12 in psoriatic plaques and non-lesional skin to further explore their role in psoriasis.

## 6. Conclusions

In this study, we investigated plasma concentrations of gal-1, gal-2 and gal-12 in psoriasis, and we are the first to report on the potential involvement of the latter two in this dermatosis. Our study revealed that the plasma gal-1, 2 and 12 in psoriatics are significantly higher than in patients without dermatoses. Given the involvement of these galectins in metabolic disorders reported in previous studies and that all of the investigated galectins were significantly higher in the plasma of patients compared to controls, this could indicate their potential engagement in metabolic complications in psoriasis. Gal-2 could be perhaps further investigated as a marker of metabolically induced inflammation in psoriasis and gal-1 and gal-12 as predictors of renal impairment in psoriatics due to metabolic disorders. Potentially, gal-12 could be considered in the future as a marker of carbohydrate metabolism disorders in psoriatics.

## Figures and Tables

**Table 1:** Basic characteristics of the patients and the control group.

Parameter	Controls (n = 30)	Psoriasis (n = 60)
Sex (M:F)	20:10	39:21 NS
Age [years]	47 ± 2.5	49 ± 2.3 NS
Height [cm]	1.7 ± 0.01	1.7 ± 0.01 NS
Weight [kg]	78 ± 3	84 ± 2 NS
BMI	25.7 ± 0.77	27.6 ± 0.8 NS

NS, non-significant.

## Data Availability

Data available upon request from the Authors.
